# Supplementary material for: Prevalence of Emergent Dolutegravir Resistance Mutations in People Living with HIV: A Rapid Scoping Review
Source: Viruses. 2024 Mar 4;16(3):399. doi: 10.3390/v16030399 (PMC10975848; doi:10.3390/v16030399)
Supplement: Supplementary file 1 [file viruses-16-00399-s001.zip › SupplementaryMaterials_Jan23.pdf]

Table S1. Virological Failure (VF) and Prevalence of Emergent INSTI-Associated DRMs in Cohorts of ART-Naïve PLWH Receiving DTG Plus Two NRTIs

Author / Year	Region/ Country	Population	DTG-containing regimen	# PLWH	# (%) with VF[1]	# (%) undergoing GRT	# (%) with INSTI DRMs
Cohort studies							
Rhee 2020 [1]	U.S. 2014-2017	Adults	DTG + 2 NRTIs	355	After a median of 26M, 6.2% had VF defined as a single VL ≥200.	5 (1.4%)	0 (0%)
Armenia 2020 [2]	Italy 2014-2017	Adults	DTG + 2 NRTIs	269	At 12M, ~3.3% had VF defined as a confirmed VL ≥50.	4 (1.5%)	0 (0%)
Ntamatungiro 2023 [3]	Tanzania 2019-2020 (KIULARCO)	Adults	DTG + TDF/3TC	436	At 12M, 6.9% had VF defined as a confirmed VL ≥1000.	2 (0.5%)	0 (0%)
Mondi 2019 [4]	Italy 2015-2017 (ICONA)	Adults	DTG + 2 NRTIs	882	After a median of 9M, ~2.6% had VF defined as a confirmed VL ≥50.	4 (0.5%)	0 (0%)
Suárez-García 2023 [5]	Spain 2018-2021 (CORIS)	Adults	DTG + 2NRTIs	537	At 12M, 10/339 (2.9%) had VF defined as a confirmed VL ≥50.	Not reported	0 (0%)
Grayhack 2018 [6][2]	U.S. 2015-2018	Pregnant women	Not reported	66	At delivery, 13/57 (22.8%) had VF defined as a single VL ≥20.	Not reported	0 (0%)
Chin 2020 [7]	Korea 2014-2017	Adults	DTG + ABC/3TC	153	After a median of 2M, 0% had VF defined as a confirmed VL ≥200.	Not applicable	Not applicable
Cross-sectional GRT studies of PLWH with VF on a DTG-containing regimen							
Diaz 2023 [8]	Brazil 2017-2018	Adults	DTG + TDF/3TC	Not known	Not reported	113	7 (6.2%): R263K (n=4); G118R; E138A; G140R

Footnotes: [1]The % with VF is preceded by '~' when the total number of individuals evaluated included some PLWH who did not receive DTG plus 2 NRTIs, most commonly DTG plus 3TC. [2]42.4% on DTG prior to pregnancy, 24.2% initiated ART with DTG during pregnancy, 33.3% switched to DTG during pregnancy. Abbreviations: 3TC – lamivudine; ABC – abacavir; ART – antiretroviral therapy; DRMs – drug-resistance mutations; DTG – dolutegravir; GRT – genotypic resistance testing; INSTI – integrase strand transfer inhibitor; M – months; NRTIs – nucleoside RT inhibitors; PLWH – people living with HIV; TDF – tenofovir disoproxil fumarate; VF – virological failure; VL – virus load as plasma HIV RNA copies/ml.

Table S2. Virological Failure (VF) and Emergent INSTI-Associated DRMs in Cohorts of ART-Naïve PLWH Receiving DTG Plus 3TC

Author / Year[1]	Region / Country	Population[2]	# PLWH	# (%) with VF at 12 months[3]	# (%) Undergoing GRT	# (%) with INSTI DRMs
Suárez-García 2023 [5]	Spain 2018-2021	Adults	401	3 (0.7%)	1 (0.2%)	0 (0%)
Cabello-Ubeda 2022 [9]	Spain ≤2020	Adults	135	1 (0.7%)	1 (0.7%)	0 (0%)
Li 2022 [10]	China 2020-2022	Adults	45	0 (0%)	Not applicable	Not applicable

<u>**Footnotes:**</u> [1]The study by Suárez-García et. al. also described a cohort of 282 individuals receiving DTG plus TDF/FTC and 255 individuals receiving DTG plus ABC/3TC (see Table S1). [2]Baseline GRT was performed in 165 (41.1%) of individuals in the study by Suárez-García et. al., 38 (28.1%) of individuals in the study by Cabello-Ubeda et. al., and all of the individuals in the study by Li et. al. One individual in the study by Suárez-García et. al. and two individuals in the study by Cabello-Ubeda et. al. had a baseline M184 mutation and were changed to a different regimen. No baseline NRTI DRMs were reported in the study by Li et. al. [3]VF was defined as a confirmed VL ≥50 or a single VL ≥1000 in each study after 48 weeks. In the study by Suárez-García et. al., 22 individuals (5.5%) discontinued therapy for reasons other than author-defined VF. In the study by Cabello-Ubeda et. al., 19 individuals (14.1%) discontinued therapy for reasons other than author-defined VF or were lost to follow-up. <u>Abbreviations:</u> 3TC – lamivudine; ABC – abacavir; ART – antiretroviral therapy; DRMs – drug-resistance mutations; FTC – emtricitabine; GRT – genotypic resistance testing; INSTI – integrase strand transfer inhibitor; PLWH – people living with HIV; VF – virological failure; VL – virus load as plasma HIV RNA copies/ml.

Table S3 Virological Failure (VF) and Emergent INSTI-Associated DRMs in Cohort Studies Describing the Use of DTG Plus Two NRTIs in PLWH With Previous VF Who Were Not Uniformly Virologically Suppressed at DTG Initiation

Author / Year	Region / Country	Population	ART History	Pre-Switch VL	# PLWH	DTG ART	# (%) with VF	# (%) Undergoing GRT	# (%) with INSTI DRMs
Upper-income country cohort studies									
Lepik 2017 [11][1]	Canada (2014-2015)	Adults	NNRTI and/or PI-containing ART; 15% baseline NRTI DRMs.	~70% had VL <50	252	DTG +2 NRTIs	At 12M, ~17% had VF defined as a confirmed VL ≥50 or DTG discontinuation.	3 (1.2%)	2 (0.8%): R263K (2)
Sorstedt 2018 [12][2]	Sweden (≤2017)	Adults	Variable; 21% INSTI-experienced; Baseline NRTI DRMs was an inclusion criterion; 37% had M184VI.	75% had VL <50	122	DTG + 2 NRTIs	After a median of 18M, 3.3% had VF defined as a single VL ≥200.	4 (3.3%)	0 (0%)
Low- and middle-income country cohort studies									
Semengue 2023 [13]	Cameroon 2021	Adults	TDF/3TC/EFV	90% had a "detectable" VL	139	DTG + TDF/3TC	At 14M, 7.9% had VF defined as VL ≥1000	11 (7.9%)	0 (0%)
Brown 2022 [14]	Lesotho 2020 (DO-REAL study)	Adults	Dual NRTI / NNRTI	Of 96% with available VL: 96% had VL <100	1225	DTG + TDF/3TC	After a median of 4M, 95% had an available VL and 1% had VF defined as VL ≥1000	7 (0.6%)	0 (0%)
Schramm 2022 [15]	Malawi 2019 (MSF program)	Adults	Dual NRTI / NNRTI	95% had VL <50	1892	DTG + TDF/3TC	At 18M, 10% (7/69) of those who were viremic before TDF/3TC/DTG developed VF defined as VL ≥50	Not reported	2 individuals with baseline dual TDF/3TC resistance developed INSTI DRMs: R263K; G118R

Cross-sectional GRT studies of PLWH with VF on a DTG-containing regimen

Van Oosterhout 2022 [16][3]	Malawi 2020-2021 (National HIV Treatment Program)	Adults / Children / Adolescents	Naïve, 1st-line ART, 2nd-line ART	Not reported	NR	Presumed DTG + 2 NRTIs	6462 (~8%) individuals had VF defined as a single VL ≥1000	27 (0.4%) of those with VF	8 samples had INSTI DRMs: R263K (3); R263K, E157Q (2); R263K, M50I; R263K+5 accessory DRMs; H51Y, S147G
Abdullahi 2023 [17][4]	Nigeria 2021	Adults	Naïve, 1st-line ART, 2nd-line ART	Not reported	4263	DTG + TDF/3TC	281 (6.7%) had VF defined as a single VL ≥1000.	33 (0.8%)	1 sample had INSTI DRMs: T66A, G118R, E138K, R263K
Kamori 2023 [18][5]	Tanzania 2020	Adults / Children	1st-line ART, 2nd-line ART	Not reported	NR	Presumed DTG + 2 NRTIs	84 (5.0%) had VF defined as a single VL ≥1000.	84 (5.0%)	4 samples had INSTI DRMs: R263K; G118R; T66A, G118R, E138K; E138K, G140A, Q148K
Khamadi 2023 [19][6]	Tanzania 2019-2021	Children / Adolescents	1st-line ART; 2nd-line ART	87% <1000	502	DTG + 2 NRTIs	64 (12.7%) of individuals had VF defined as a single VL ≥1000	~40 (~8.0%)	3 samples had INSTI DRMs: R263K (3)
Bwire 2023 [20]	Tanzania 2023	Adults	Naïve, 1st-line ART, 2nd-line ART	Not reported	600	DTG + TDF/3TC	33 (5.5%) of individuals had VF defined as a single VL ≥1000	30 (5.0%)	3 samples had INSTI DRMs: G118R, E138K; E138K, G140A, Q148K; T66I, G118R, E138K

Footnotes: [1]This study also included individuals (21% of the total) who had been INSTI-experienced. The "~" indicates that the precise proportions with baseline VS and with VF following DTG were not available for the subset who were INSTI experienced. [2]One individual had VF while receiving raltegravir prior to receiving DTG but had not developed INSTI DRMs; [3]87 applications were submitted for sequencing; 33 samples were selected, and 27 were successfully sequenced. [4]Sample collection was successful in 61 individuals and 33 of these were successfully sequenced. [5]The study included 367 individuals who did not receive a DTG-containing regimen. However, because GRT was successful in

98.5% of individuals with VF in the study, we estimated that all had undergone GRT. [6]A random selection of 707 children and adolescents on first- or second-line ART for ≥6 months were selected of whom 71.0% were receiving DTG plus 2 NRTIs. The number undergoing GRT was estimated from the 63% rate of successful sequencing for the complete cohort. Abbreviations: 1st-line ART – generally refers to a nonnucleoside RT inhibitor plus 2 NRTIs; 2nd-line ART – generally refers to a protease-inhibitor containing regimen; 3TC – lamivudine; ART – antiretroviral therapy; DRMs – drug-resistance mutations; GRT – genotypic resistance testing; INSTI – integrase strand transfer inhibitor; NR – not reported; NRTIs – nucleoside reverse transcriptase inhibitors; PLWH – people living with HIV; TDF – tenofovir disoproxil fumarate; VF – virological failure; VL – virus load as plasma HIV RNA copies/ml; VS – virological suppression.

Table S4. Virological Failure (VF) and Emergent INSTI-Associated DRMs in Cohorts of ART-Experienced PLWH with VS Receiving DTG Plus Two NRTIs

Author / Year[1]	Region/ Country	ART History[2]	# PLWH	DTG ART	# (%) with VF[3]	# (%) Undergoing GRT	# (%) With INSTI DRMs
Olearo 2019 [21]	Europe	VS (median >84M); 19% had h/o INSTI; >30% h/o VF; 8.4% had h/o M184VI	1626	DTG + ABC/3TC	21 (1.3%) (median 9M)	6 (0.4%)	0 (0%)
Borghetti 2022 [22]	Italy	VS (median ~48M); INSTI history not reported; 11% h/o VF; 12% h/o M184VI	424	DTG + ABC/3TC (72%) or TDF/FTC (28%)	31 (7.3%) (median 21M)	6 (1.4%)	0 (0%)
Jary 2020 [23]	France	VS (median 36M); INSTI-naïve; ~82% had h/o VF and M184VI	154	DTG + ABC/3TC	0 (0%) (at 12M)	3 (2.0%)	0 (0%)

Footnotes: [1]The study by Borghetti et. al. also included cohorts with virological suppression (VS) that received DTG/3TC and was therefore also in Table S5. [2]M (months). With the exception of Olearo et al., each study explicitly reported that no individual experienced VF while receiving an INSTI. [3]VF was defined as a confirmed VL ≥50 or a single VL ≥200 for Jary et. al., and Borghetti et. al. and as a confirmed VL ≥50 or as single VL ≥50 at the last time point for Olearo et. al. M184VI did not appear to increase the risk of VF in the study by Olearo et. al. Abbreviations: 3TC – lamivudine; ABC – abacavir; ART – antiretroviral therapy; DRMs – drug-resistance mutations; DTG – dolutegravir; FTC – emtricitabine; GRT – genotypic resistance testing; h/o – history of; INSTI – integrase strand transfer inhibitor; M – months; PLWH – people living with HIV; TDF – tenofovir disoproxil fumarate; VF – virological failure; VS – virological suppression.

Table S5. Virological Failure (VF) and Emergent INSTI-Associated DRMs in Cohorts of ART-Experienced PLWH with VS Receiving DTG Plus a 2nd ARV

Author/Year[1]	Countries / Years	ART History[2]	# PLWH	DTG ART	# (%) with VF[3]	# (%) Undergoing GRT	# (%) with INSTI DRMs
Palmier 2022 [24]	Spain 2020-2019	VS (median duration not reported); 27.1% had h/o VF; 70.7% INSTI-experienced; 4.7% had historical M184VI.	358	DTG/3TC	After a mean of 36M, 3.6% had VF defined as VL ≥50.	9 (2.5%)	1 (0.3%); R263K
Bowman 2023 [25][4]	UK 2015-2021	VS (median duration not reported); 3.7% had VL ≥50, % with h/o VF not reported; 14.3% INSTI-experienced.	552	DTG/XTC (86.6%) DTG/RPV (13.4%)	After a median of 11M, 5/460 (1.1%) on DTG/3TC had VF. After a median of 28M, 1/74 (1.4%) on DTG/RPV had VF. VF was defined as a confirmed VL ≥200.	5 (0.9%)	1 (0.2%) individual on DTG/3TC: T66A, G118R, E138K
Knobel 2023 [26][5]	Spain 2019-2022	VS (median duration not reported); 42% had been on a DTG-containing regimen; 12% had received a first-generation INSTI; % with h/o VF not reported.	358	DTG/3TC	After a median of ~36M, 1.1% had VF defined as a confirmed VL ≥200.	4 (1.1%)	1 (0.3%); G118R, R263K
Baldin 2019 [27]	Italy (≤2019)	VS (median 96M); 43.4% had h/o VF; 19.5% INSTI-experienced; 9.0% had historical M184VI.	221	DTG/3TC	After a median of 25M, 3.2% had VF defined as a confirmed VL ≥50 or a single VL ≥1000.	7 (3.2%)	0 (0%)
Borghetti 2022 [22]	Italy 2014-2020	VS (median 48M); 10.8% had h/o VF; INSTI history not reported; 3.9% had historical M184VI.	204	DTG/3TC	After a median of 20M, 4.9% had VF defined as a confirmed VL ≥50 or a single VL ≥200.	1 (0.5%)	(0%)
Calza 2020 [28]	Italy 2016-2018	VS (median 41M); No h/o VF on INSTI or XTC-containing regimen; 80.0% INSTI-experienced.	59	DTG/3TC	At 12M, 3.4% had VF defined as a single VL ≥20.	2 (3.4%)	0 (0%)
Calza 2023 [29]	Italy 2018-2020	Age > 65; VS (median 71M); No h/o VF; INSTI-naïve.	72	DTG/3TC	At 12M, 4.2% had VF defined as a single VL ≥20.	3 (4.2%)	0 (0%)
Cicccullo 2021 [30]	Italy 2016-2021	VS (median 29M); 22.3% had h/o VF; 27.5% INSTI-experienced; 4.2% had historical M184VI.	785	DTG/3TC	After a mean of 30M, 2.3% had VF defined as a confirmed VL ≥50 or a single VL ≥1000.	Not reported	0 (0%)
Cicccullo 2023 [31]	Italy 2015-2021	VS (median 80M); 32.7% had h/o VF; 27.3% INSTI-experienced.	592	DTG/3TC (51.7%)	After a median of 25M, 2.6% on DTG/3TC and after a median of	11 (0.2%)	0 (0%)

				DTG/RPV (48.3%)	28M, 1.0% on DTG/RPV had VF defined as a confirmed VL ≥50 or a single VL ≥1000.		
Maggiolo 2022 [32]	Italy 2015-2017	VS (median 75M); % with h/o VF not reported; 22.5% INSTI-experienced; 0% had historical M184VI.	218	DTG/3TC	After a mean of 64M, 0% had VF defined as a confirmed VL ≥50.	Not applicable	Not applicable
Lee 2022 [33]	Korea 2020-2022	VS (median duration not reported); % with h/o VF not reported; 91% INSTI-experienced; 2.0% had historical M184VI.	131	DTG/3TC	At 12M, 0% had VF defined as a VL ≥1000.	Not applicable	Not applicable
Ergen 2022 [34]	Turkey 2016-2021	VS (median duration not reported); No h/o VF; 80.9% INSTI-experienced.	63	DTG/3TC	After a median of 10M, 0% had VF defined as VL ≥200.	Not applicable	Not applicable
Buzon 2023 [35]	Spain 2020-2021	VS (median duration not reported); % with h/o VF not reported; 44.5% INSTI-experienced.	1032	DTG/3TC	At 12M, 2.5% of 763 had VF defined as a single VL ≥50.	Not reported	0 (0%)
Troya 2022 [36]	Spain 2018-2019 (DORIPEX)	VS (median duration not reported); % with h/o VF not reported; 41.0% INSTI-experienced.	524	DTG/RPV	At 12M, 0.6% had VF defined as a single VL ≥50.	Not reported	0 (0%)
Gantner 2017 [37]	France 2014-2015	VS (median 120M); 52% had h/o VF; 59% INSTI-experienced.	152	DTG/RPV	After a median of 9M, 2.0% had VF defined as a confirmed VL ≥50 or a single VL ≥1000.	2 (1.3%)	0 (0%)
Casado 2019 [38]	Spain 2015-2017	VS (median 52M); % with h/o VF not reported; 15% INSTI-experienced.	102	DTG/RPV	At 12M, 1.0% had VF defined as a confirmed VL ≥50.	1 (1.0%)	0 (0%)
Poliseno 2023 [39]	Italy 2020-2021	VS (median 96M); 66% INSTI-experienced; 6 individuals (13%) had detectable VL at the time DTG/DOR was begun	43	DTG/DOR	At 12M, 2.3% had VF defined as a confirmed VL ≥50 or a single VL ≥200.	1 (2.3%)	0 (0%)
Castagna 2019 [40]	Italy 2014-2018	VS (median 44M); % with h/o VF not reported; 49.1% INSTI-experienced	151	DTG/ATV	After a median of 15M, 1.3% had VF defined as a confirmed VL ≥50.	1 (0.7%)	0 (0%)

Footnotes: [1]Borghetti et. al. also included a cohort of individuals with VS that received DTG plus 2 NRTIs and is summarized in Table S4. Part of the Maggiolo cohort was reported in an earlier publication [41]. [2]INSTI-experienced includes persons who received either a first-generation or second-generation INSTI. [3]Higher rate of VF (but not INSTI DRMs) occurred in individuals with baseline M184VI in certain cohorts. In Cicculo et. al., 2021, this included the subgroup with VS for less than 88 months. In Baldin et. al., this included the subgroup with VS for less than 96 months. In Santoro et. al., this included those with VS for less than 42 months. [4]Included two individuals with baseline INSTI DRMs (F121Y, N155H), neither of whom developed VF; [5]The one individual who developed INSTI DRMs had a history of receiving an elvitegravir-containing regimen but had not developed VF on that regimen. Abbreviations: 3TC – lamivudine; ABC – abacavir; ART – antiretroviral therapy; ARV – antiretroviral; ATV – atazanavir; DOR – doravirine; DRMs – drug-resistance mutations; DTG – dolutegravir; FTC – emtricitabine; GRT – genotypic resistance testing; h/o – history of; INSTI – integrase strand

transfer inhibitor; M – months; PLWH – people living with HIV; RPV – rilpivirine; TDF – tenofovir disoproxil fumarate; VF – virological failure; VS – virological suppression; XTC – 3TC or FTC.

Table S6. Virological Failure (VF) and Emergent INSTI-Associated DRMs in Cohorts of ART-Experienced PLWH with VS Receiving DTG Monotherapy

Author/Year[1]	Countries / Years	ART History	# PLWH	# (%) VF	# (%) Undergoing GRT at VF	# (%) with INSTI DRMs[2]
Rojas 2016 [42]	Spain 2014-2015	VS (median 96M); received a median 9 different ART regimens; 6% INSTI-experienced; no history of VF on an INSTI regimen.	31	At 6M, 1 individual (3.2%) had VF defined as a confirmed VL ≥50.	1 (3.2%)	1 (32%); G118R
Oldenbuettal 2017 [43]	Germany 2014-2016	VS (median duration not reported); 61% INSTI-experienced; no history of VF on an INSTI regimen.	31	At 6M, 1 individual (3.2%) had VF defined as a confirmed VL ≥50.	1 (3.2%)	1 (3.2%); G140S, Q148H
Tebano 2020 [44]	France 2014-2018	VS (median duration 70M); 39% INSTI-experienced; no history of VF on an INSTI regimen.	61	After a median duration of 24M, 4.9% had VF defined as a confirmed VL ≥50 or a single VL ≥200.	3 (4.9%)	3 (4.9%) (4.9%); E138K, G140A, Q148R; E92Q; N155H

Footnotes: [1]Tebano et. al. describes the longer term follow-up of a cohort originally reported by Katalama et. al. in 2016 [45]. Two additional cohorts included less than 30 individuals of whom none developed INSTI DRMs [46,47]. [2]In the study by Rojas et. al., G118R was detected by next-generation sequencing of a peripheral blood mononuclear sequence in 7% of sequence reads. Abbreviations: ART – antiretroviral therapy; DRMs – drug-resistance mutations; DTG – dolutegravir; GRT – genotypic resistance testing; INSTI – integrase strand transfer inhibitor; M – months; VF – virological failure; VL – virus load as plasma HIV RNA copies/ml; VS – virological suppression.

References

1. Rhee, S.-Y.; Clutter, D.; Hare, C.B.; Tchakoute, C.T.; Sainani, K.; Fessel, W.J.; Hurley, L.; Slome, S.; Pinsky, B.A.; Silverberg, M.J.; et al. Virological Failure and Acquired Genotypic Resistance Associated With Contemporary Antiretroviral Treatment Regimens. *Open Forum Infectious Diseases* **2020**, *7*, ofaa316, doi:10.1093/ofid/ofaa316.
2. Armenia, D.; Bouba, Y.; Gagliardini, R.; Gori, C.; Bertoli, A.; Borghi, V.; Gennari, W.; Micheli, V.; Callegaro, A.P.; Gazzola, L.; et al. Evaluation of Virological Response and Resistance Profile in HIV-1 Infected Patients Starting a First-Line Integrase Inhibitor-Based Regimen in Clinical Settings. *Journal of Clinical Virology* **2020**, *130*, 104534, doi:10.1016/j.jcv.2020.104534.
3. Ntamatungiro, A.J.; Eichenberger, A.; Okuma, J.; Vanobberghen, F.; Ndege, R.; Kimera, N.; Francis, J.M.; Kagura, J.; Weisser, M.; for the Kilombero and Ulanga Antiretroviral Cohort (KIULARCO) Study Group Transitioning to Dolutegravir in a Programmatic Setting: Virological Outcomes and Associated Factors Among Treatment-Naive Patients With HIV-1 in the Kilombero and Ulanga Antiretroviral Cohort in Rural Tanzania. *Open Forum Infectious Diseases* **2023**, *10*, ofad321, doi:10.1093/ofid/ofad321.
4. Mondi, A.; Cozzi-Lepri, A.; Tavelli, A.; Rusconi, S.; Vichi, F.; Ceccherini-Silberstein, F.; Calcagno, A.; De Luca, A.; Maggiolo, F.; Marchetti, G.; et al. Effectiveness of Dolutegravir-Based Regimens as Either First-Line or Switch Antiretroviral Therapy: Data from the Icona Cohort. *Journal of the International AIDS Society* **2019**, *22*, e25227, doi:10.1002/jia2.25227.
5. Suárez-García, I.; Alejos, B.; Hernando, V.; Viñuela, L.; Vera García, M.; Rial-Crestelo, D.; Pérez Elías, M.J.; Albendín Iglesias, H.; Peraire, J.; Tiraboschi, J.; et al. Effectiveness and Tolerability of Dolutegravir/Lamivudine for the Treatment of HIV-1 Infection in Clinical Practice. *Journal of Antimicrobial Chemotherapy* **2023**, *78*, 1423–1432, doi:10.1093/jac/dkad102.
6. Grayhack, C.; Sheth, A.; Kirby, O.; Davis, J.; Sibliss, K.; Nkwihoreze, H.; Aaron, E.; Alleyne, G.; Laguerre, R.; Rana, A.; et al. Evaluating Outcomes of Mother–Infant Pairs Using Dolutegravir for HIV Treatment during Pregnancy. *AIDS* **2018**, *32*, 2017, doi:10.1097/QAD.0000000000001931.
7. Chin, B.S.; Lee, J.H.; Kim, G. Similar Durability of Two Single Tablet Regimens, Dolutegravir/Abacavir/Lamivudine and Elvitegravir/Cobicistat/Tenofovir/Emtricitabine: Single Center Experience. *J Korean Med Sci* **2020**, *35*, e235, doi:10.3346/jkms.2020.35.e235.
8. Diaz, R.S.; Hunter, J.R.; Camargo, M.; Dias, D.; Galinskas, J.; Nassar, I.; De Lima, I.B.; Caldeira, D.B.; Sucupira, M.C.; Schechter, M. Dolutegravir-Associated Resistance Mutations after First-Line Treatment Failure in Brazil. *BMC Infect Dis* **2023**, *23*, 347, doi:10.1186/s12879-023-08288-8.
9. Cabello-Ubeda, A.; Quirós, J.C.L.B. de; Carbonero, L.M.; Sanz, J.; Vergas, J.; Mena, Á.; Torralba, M.; Segurado, M.H.; Pinto, A.; Tejerina, F.; et al. 48-Week Effectiveness and Tolerability of Dolutegravir (DTG) + Lamivudine (3TC) in Antiretroviral-Naïve Adults Living with HIV: A Multicenter Real-Life Cohort. *PLOS ONE* **2022**, *17*, e0277606, doi:10.1371/journal.pone.0277606.

10. Li, J.; Chen, D.; Wen, Z.; Du, Y.; Huang, Z.; Zhong, H.; Wang, Y.; Yin, S. Real-World Efficacy and Safety of Dolutegravir plus Lamivudine versus Tenofovir plus Lamivudine and Efavirenz in ART-Naïve HIV-1-Infected Adults. *Medicine* **2022**, *101*, e31100, doi:10.1097/MD.0000000000031100.
11. Lepik, K.J.; Harrigan, P.R.; Yip, B.; Wang, L.; Robbins, M.A.; Zhang, W.W.; Toy, J.; Akagi, L.; Lima, V.D.; Guillemi, S.; et al. Emergent Drug Resistance with Integrase Strand Transfer Inhibitor-Based Regimens. *AIDS* **2017**, *31*, 1425–1434, doi:10.1097/QAD.0000000000001494.
12. Sörstedt, E.; Carlander, C.; Flamholc, L.; Hejdeman, B.; Svedhem, V.; Sönnerborg, A.; Gisslén, M.; Yilmaz, A. Effect of Dolutegravir in Combination with Nucleoside Reverse Transcriptase Inhibitors (NRTIs) on People Living with HIV Who Have Pre-Existing NRTI Mutations. *International Journal of Antimicrobial Agents* **2018**, *51*, 733–738, doi:10.1016/j.ijantimicag.2018.01.009.
13. Semengue, E.N.J.; Fokam, J.; Etame, N.-K.; Molimbou, E.; Chenwi, C.A.; Takou, D.; Mossiang, L.; Meledie, A.P.; Yagai, B.; Nka, A.D.; et al. Dolutegravir-Based Regimen Ensures High Virological Success despite Prior Exposure to Efavirenz-Based First-LINE ART in Cameroon: An Evidence of a Successful Transition Model. *Viruses* **2023**, *15*, 18, doi:10.3390/v15010018.
14. Brown, J.A.; Nsakala, B.L.; Mokhele, K.; Rakuoane, I.; Muhairwe, J.; Urda, L.; Amstutz, A.; Tschumi, N.; Klimkait, T.; Labhardt, N.D. Viral Suppression after Transition from Nonnucleoside Reverse Transcriptase Inhibitor- to Dolutegravir-Based Antiretroviral Therapy: A Prospective Cohort Study in Lesotho (DO-REAL Study). *HIV Medicine* **2022**, *23*, 287–293, doi:10.1111/hiv.13189.
15. Schramm, B.; Temfack, E.; Descamps, D.; Nicholas, S.; Peytavin, G.; Bitilinyu-Bangoh, J.E.; Storto, A.; Lê, M.P.; Abdi, B.; Ousley, J.; et al. Viral Suppression and HIV-1 Drug Resistance 1 Year after Pragmatic Transitioning to Dolutegravir First-Line Therapy in Malawi: A Prospective Cohort Study. *Lancet HIV* **2022**, *9*, e544–e553, doi:10.1016/S2352-3018(22)00136-9.
16. van Oosterhout, J.J.; Chipungu, C.; Nkhoma, L.; Kanise, H.; Hosseinipour, M.C.; Sagno, J.B.; Simon, K.; Cox, C.; Hoffman, R.; Steegen, K.; et al. Dolutegravir Resistance in Malawi's National HIV Treatment Program. *Open Forum Infect Dis* **2022**, *9*, ofac148, doi:10.1093/ofid/ofac148.
17. Abdullahi, A.; Kida, I.M.; Maina, U.A.; Ibrahim, A.H.; Mshelia, J.; Wisso, H.; Adamu, A.; Onyemata, J.E.; Edun, M.; Yusuph, H.; et al. Limited Emergence of Resistance to Integrase Strand Transfer Inhibitors (INSTIs) in ART-Experienced Participants Failing Dolutegravir-Based Antiretroviral Therapy: A Cross-Sectional Analysis of a Northeast Nigerian Cohort. *Journal of Antimicrobial Chemotherapy* **2023**, *78*, 2000–2007, doi:10.1093/jac/dkad195.
18. Kamori, D.; Barabona, G.; Rugemalila, J.; Maokola, W.; Masoud, S.S.; Mizinduko, M.; Sabasaba, A.; Ruhago, G.; Sambu, V.; Mushi, J.; et al. Emerging Integrase Strand Transfer Inhibitor Drug Resistance Mutations among Children and Adults on ART in Tanzania: Findings from a National Representative HIV Drug Resistance Survey. *Journal of Antimicrobial Chemotherapy* **2023**, *78*, 779–787, doi:10.1093/jac/dkad010.
19. Khamadi, S.A.; Bahemana, E.; Dear, N.; Mavere, C.; George, F.; Kapene, R.; Papianus, G.; Willoughby, W.; Chambers, J.; Ganesan, K.; et al. Factors Associated With Viral Suppression and Drug Resistance in Children and Adolescents Living With HIV in Care and

Treatment Programs in Southern Tanzania. *Journal of the Pediatric Infectious Diseases Society* **2023**, *12*, 353–363, doi:10.1093/jpids/piad040.
20. Bwire, G.M.; Aiko, B.G.; Mosha, I.H.; Kilapilo, M.S.; Mangara, A.; Kazonda, P.; Swai, J.P.; Swalehe, O.; Jordan, M.R.; Vercauteren, J.; et al. High Viral Suppression and Detection of Dolutegravir-Resistance Associated Mutations in Treatment-Experienced Tanzanian Adults Living with HIV-1 in Dar Es Salaam. *Sci Rep* **2023**, *13*, 20493, doi:10.1038/s41598-023-47795-1.
21. Olearo, F.; Nguyen, H.; Bonnet, F.; Yerly, S.; Wandeler, G.; Stoeckle, M.; Cavassini, M.; Scherrer, A.; Costagiola, D.; Schmid, P.; et al. Impact of the M184V/I Mutation on the Efficacy of Abacavir/Lamivudine/Dolutegravir Therapy in HIV Treatment-Experienced Patients. *Open Forum Infectious Diseases* **2019**, *6*, ofz330, doi:10.1093/ofid/ofz330.
22. Borghetti, A.; Alkhatib, M.; Dusina, A.; Duca, L.; Borghi, V.; Zazzi, M.; Di Giambenedetto, S. Virological Outcomes with Dolutegravir plus Either Lamivudine or Two NRTIs as Switch Strategies: A Multi-Cohort Study. *Journal of Antimicrobial Chemotherapy* **2022**, *77*, 740–746, doi:10.1093/jac/dkab429.
23. Jary, A.; Marcelin, A.-G.; Charpentier, C.; Wirden, M.; Lê, M.P.; Peytavin, G.; Descamps, D.; Calvez, V. M184V/I Does Not Impact the Efficacy of Abacavir/Lamivudine/Dolutegravir Use as Switch Therapy in Virologically Suppressed Patients. *Journal of Antimicrobial Chemotherapy* **2020**, *75*, 1290–1293, doi:10.1093/jac/dkaa019.
24. Palmier, E.; De Miguel, R.; Montejano, R.; Busca, C.; Micán, R.; Ramos, L.; Cadiñanos, J.; Serrano, L.; Bernardino, J.I.; Pérez-Valero, I.; et al. Three-Year Efficacy of Switching to Dolutegravir plus Lamivudine: A Real-World Study. *HIV Medicine* **2023**, *n/a*, doi:10.1111/hiv.13500.
25. Bowman, C.; Ambrose, A.; Kanitkar, T.; Flores, K.; Simoes, P.; Hart, J.; Hunter, A.; Akodu, J.; Barber, T.J. Real World Use of Dolutegravir Two Drug Regimens. *AIDS* **2023**, *37*, 785, doi:10.1097/QAD.0000000000003480.
26. Knobel, H.; Cañas-Ruano, E.; Guelar, A.; Knobel, P.; Villar-García, J.; González-Mena, A.; Canepa, C.; Arrieta-Aldea, I.; Marcos, A.; Abalat-Torrres, A.; et al. Switching to Dolutegravir/Lamivudine or Bictegravir/Emtricitabine/Tenofovir Alafenamide. A Comparative Real-World Study. *HIV Research & Clinical Practice* **2023**, *24*, 2239564, doi:10.1080/25787489.2023.2239564.
27. Baldin, G.; Ciccullo, A.; Borghetti, A.; Di Giambenedetto, S. Virological Efficacy of Dual Therapy with Lamivudine and Dolutegravir in HIV-1-Infected Virologically Suppressed Patients: Long-Term Data from Clinical Practice. *Journal of Antimicrobial Chemotherapy* **2019**, *74*, 1461–1463, doi:10.1093/jac/dkz009.
28. Calza, L.; Colangeli, V.; Borderi, M.; Testi, D.; Granozzi, B.; Bon, I.; Re, M.C.; Viale, P. Simplification to Dual Therapy Containing Lamivudine and Raltegravir or Dolutegravir in HIV-Infected Patients on Virologically Suppressive Antiretroviral Therapy. *Journal of Antimicrobial Chemotherapy* **2020**, *75*, 3327–3333, doi:10.1093/jac/dkaa319.
29. Calza, L.; Colangeli, V.; Legnani, G.; Cretella, S.; Bon, I.; Viale, P. EFFICACY AND SAFETY OF SWITCHING TO DOLUTEGRAVIR/LAMIVUDINE IN VIROLOGICALLY SUPPRESSED PEOPLE LIVING WITH HIV-1 AGED OVER 65 YEARS. *AIDS Res Hum Retroviruses* **2023**, doi:10.1089/AID.2023.0046.

30. Ciccullo, A.; Borghi, V.; Giacomelli, A.; Cossu, M.V.; Sterrantino, G.; Latini, A.; Giacometti, A.; De Vito, A.; Gennari, W.; Madeddu, G.; et al. Five Years With Dolutegravir Plus Lamivudine as a Switch Strategy: Much More Than a Positive Finding. *JAIDS Journal of Acquired Immune Deficiency Syndromes* **2021**, *88*, 234, doi:10.1097/QAI.0000000000002787.
31. Ciccullo, A.; Baldin, G.; Borghi, V.; Cossu, M.V.; Giacomelli, A.; Lagi, F.; Farinacci, D.; Iannone, V.; Passerotto, R.A.; Capetti, A.; et al. Analysing the Efficacy and Tolerability of Dolutegravir plus Either Rilpivirine or Lamivudine in a Multicentre Cohort of Virologically Suppressed PLWHIV. *Journal of Antimicrobial Chemotherapy* **2023**, *78*, 117–121, doi:10.1093/jac/dkac362.
32. Maggiolo, F.; Gulminetti, R.; Pagnucco, L.; Digaetano, M.; Cervo, A.; Valenti, D.; Callegaro, A.; Mussini, C. Long-Term Outcome of Lamivudine/Dolutegravir Dual Therapy in HIV-Infected, Virologically Suppressed Patients. *BMC Infectious Diseases* **2022**, *22*, 782, doi:10.1186/s12879-022-07769-6.
33. Lee, K.H.; Kim, J.; Lee, J.A.; Kim, C.H.; Ahn, J.Y.; Jeong, S.J.; Ku, N.S.; Choi, J.Y.; Yeom, J.-S.; Song, Y.G.; et al. Real-World Effectiveness, Tolerability, and Safety of Dolutegravir/Lamivudine in Korea. *Viruses* **2022**, *14*, 2558, doi:10.3390/v14112558.
34. Ergen, P.; Bektas, B.; Aydın, Ö.; Keskin, H.; Üçışık, A.C.; Karadağ, F.Y.; Cağ, Y. Evaluation of Treatment Efficacy after Switching to Dolutegravir-Lamivudine Dual Therapy in People Living with HIV. *African Health Sciences* **2022**, *22*, 426–435, doi:10.4314/ahs.v22i3.46.
35. Buzón, L.; Dueñas, C.; Pedrero, R.; Iribarren, J.A.; de los Santos, I.; Díaz de Santiago, A.; Morán, M.Á.; Pousada, G.; Moreno, E.; Ferreira, E.; et al. Dolutegravir Plus 3TC in Virologically Suppressed PLWHIV: Immunological Outcomes in a Multicenter Retrospective Cohort in Spain during the COVID-19 Pandemic. *Viruses* **2023**, *15*, 322, doi:10.3390/v15020322.
36. Troya, J.; Dueñas, C.; Irazola, I.; de los Santos, I.; de la Fuente, S.; Gil, D.; Hernández, C.; Galindo, M.J.; Gómez, J.; Delgado, E.; et al. Dolutegravir plus Rilpivirine: Benefits beyond Viral Suppression: DORIPEX Retrospective Study. *Medicine* **2022**, *101*, e29252, doi:10.1097/MD.0000000000029252.
37. Gantner, P.; Cuzin, L.; Allavena, C.; Cabie, A.; Pugliese, P.; Valantin, M.-A.; Bani-Sadr, F.; Joly, V.; Ferry, T.; Poizot-Martin, I.; et al. Efficacy and Safety of Dolutegravir and Rilpivirine Dual Therapy as a Simplification Strategy: A Cohort Study. *HIV Medicine* **2017**, *18*, 704–708, doi:10.1111/hiv.12506.
38. Casado, J.L.; Monsalvo, M.; Fontecha, M.; Vizcarra, P.; Rodriguez, M.A.; Vivancos, M.J.; Moreno, S. Dolutegravir plus Rilpivirine as Dual Regimen in Virologically Suppressed HIV-1 Infected Patients in a Clinical Setting. *HIV Research & Clinical Practice* **2019**, *20*, 64–72, doi:10.1080/15284336.2019.1628460.
39. Poliseno, M.; Mazzitelli, M.; Narducci, A.; Ferrara, S.M.; Resnati, C.; Gervasoni, C.; Cattelan, A.M.; Lo Caputo, S. Doravirine Plus Integrase Strand Transfer Inhibitors as a 2-Drug Treatment–Switch Strategy in People Living with HIV: The Real-Life DORINI Multicentric Cohort Study. *JAIDS Journal of Acquired Immune Deficiency Syndromes* **2023**, *94*, 235, doi:10.1097/QAI.0000000000003248.

40. Castagna, A.; Rusconi, S.; Gulminetti, R.; Bonora, S.; Mazzola, G.; Quiros-Roldan, M.E.; De Socio, G.V.; Ladisa, N.; Carosella, S.; Cattelan, A.; et al. Switch to Dolutegravir and Unboosted Atazanavir in HIV-1 Infected Patients with Undetectable Viral Load and Long Exposure to Antiretroviral Therapy. *AIDS* **2019**, *33*, 1256, doi:10.1097/QAD.0000000000002188.
41. Maggiolo, F.; Gulminetti, R.; Pagnucco, L.; Digaetano, M.; Benatti, S.; Valenti, D.; Callegaro, A.; Ripamonti, D.; Mussini, C. Lamivudine/Dolutegravir Dual Therapy in HIV-Infected, Virologically Suppressed Patients. *BMC Infectious Diseases* **2017**, *17*, 215, doi:10.1186/s12879-017-2311-2.
42. Rojas, J.; Blanco, J.L.; Marcos, M.A.; Lonca, M.; Tricas, A.; Moreno, L.; Gonzalez-Cordon, A.; Torres, B.; Mallolas, J.; Garcia, F.; et al. Dolutegravir Monotherapy in HIV-Infected Patients with Sustained Viral Suppression. *Journal of Antimicrobial Chemotherapy* **2016**, *71*, 1975–1981, doi:10.1093/jac/dkw078.
43. Oldenbuettel, C.; Wolf, E.; Ritter, A.; Noe, S.; Heldwein, S.; Pascucci, R.; Wiese, C.; Krosigk, A.V.; Jaegel-Guedes, E.; Jaeger, H.; et al. Dolutegravir Monotherapy as Treatment De-Escalation in HIV-Infected Adults with Virological Control: DoluMono Cohort Results. *Antiviral Therapy* **2017**, *22*, 169–172, doi:10.3851/IMP3082.
44. Tebano, G.; Soulié, C.; Schneider, L.; Blanc, C.; Agher, R.; Seang, S.; Valantin, M.A.; Palich, R.; Tubiana, R.; Peytavin, G.; et al. Long-Term Follow-up of HIV-Infected Patients on Dolutegravir Monotherapy. *Journal of Antimicrobial Chemotherapy* **2020**, *75*, 675–680, doi:10.1093/jac/dkz478.
45. Katlama, C.; Soulié, C.; Caby, F.; Denis, A.; Blanc, C.; Schneider, L.; Valantin, M.-A.; Tubiana, R.; Kirstetter, M.; Valdenassi, E.; et al. Dolutegravir as Monotherapy in HIV-1-Infected Individuals with Suppressed HIV Viraemia. *Journal of Antimicrobial Chemotherapy* **2016**, *71*, 2646–2650, doi:10.1093/jac/dkw186.
46. Gubavu, C.; Prazuck, T.; Niang, M.; Buret, J.; Mille, C.; Guinard, J.; Avettand-Fènoël, V.; Hocqueloux, L. Dolutegravir-Based Monotherapy or Dual Therapy Maintains a High Proportion of Viral Suppression Even in Highly Experienced HIV-1-Infected Patients. *Journal of Antimicrobial Chemotherapy* **2016**, *71*, 1046–1050, doi:10.1093/jac/dkv430.
47. Lattuada, E.; Lanzafame, M.; Nicolè, S.; Rigo, F.; Cucchetto, G.; Concia, E.; Vento, S. Dolutegravir Monotherapy in HIV-1-Suppressed Patients: A Feasible Regimen in Real Life. *Int J STD AIDS* **2018**, *29*, 206–207, doi:10.1177/0956462417747832.
